# Supplementary material for: Evaluating methods for B-cell clonal family assignment
Source: bioRxiv. 2024 Jun 2:2024.05.29.596491. Preprint. [Version 1] doi: 10.1101/2024.05.29.596491 (PMC11160721; doi:10.1101/2024.05.29.596491)
Supplement: Supplement 1 [file NIHPP2024.05.29.596491v1-supplement-1.pdf]

bioRxiv preprint doi: https://doi.org/10.1101/2024.05.29.596491; this version posted June 2, 2024. The copyright holder for this preprint (which was not certified by peer review) is the author/funder, who has granted bioRxiv a license to display the preprint in perpetuity. It is made available under aCC-BY 4.0 International license.

Supplementary Material

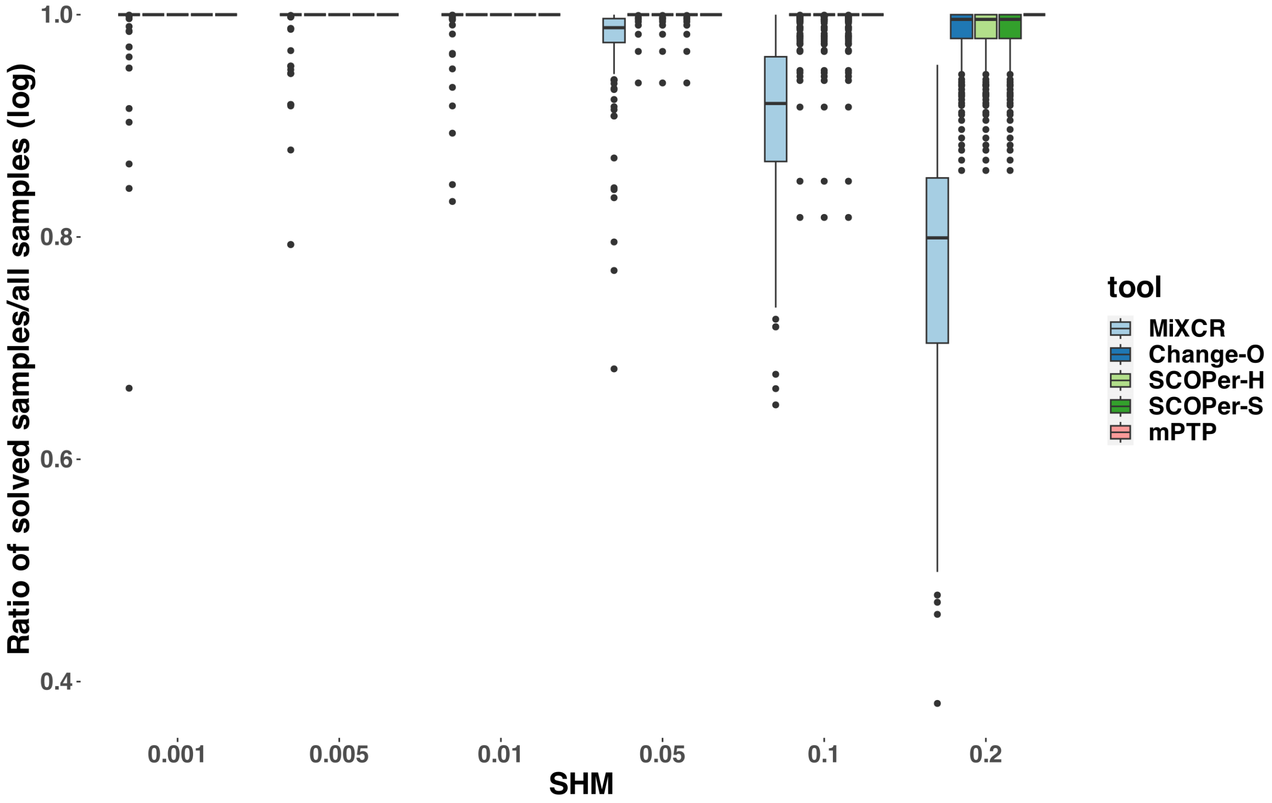

Figure S1: **Ratio of solved samples/all samples (log) across different SHM rates.** For this analysis we counted the amount of sequences that the methods assigned to a clonal family and divided that by the number of all sequences from the input.

bioRxiv preprint doi: https://doi.org/10.1101/2024.05.29.596491; this version posted June 2, 2024. The copyright holder for this preprint (which was not certified by peer review) is the author/funder, who has granted bioRxiv a license to display the preprint in perpetuity. It is made available under aCC-BY 4.0 International license.

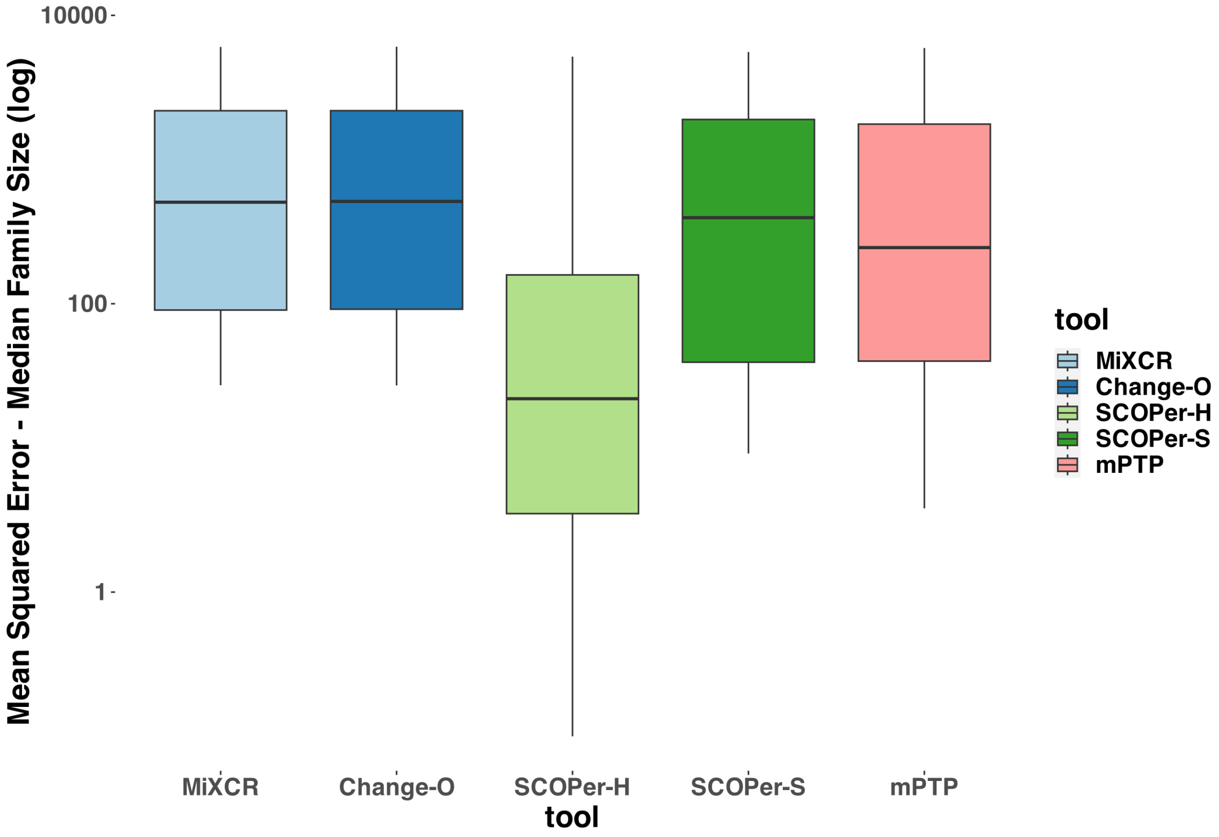

Figure S2: **MSE of the median family size.** For this analysis we removed singletons. We calculated the median family size of the true clonal families and compared them to the median family size of the derived clonal families for each method.

bioRxiv preprint doi: https://doi.org/10.1101/2024.05.29.596491; this version posted June 2, 2024. The copyright holder for this preprint (which was not certified by peer review) is the author/funder, who has granted bioRxiv a license to display the preprint in perpetuity. It is made available under aCC-BY 4.0 International license.

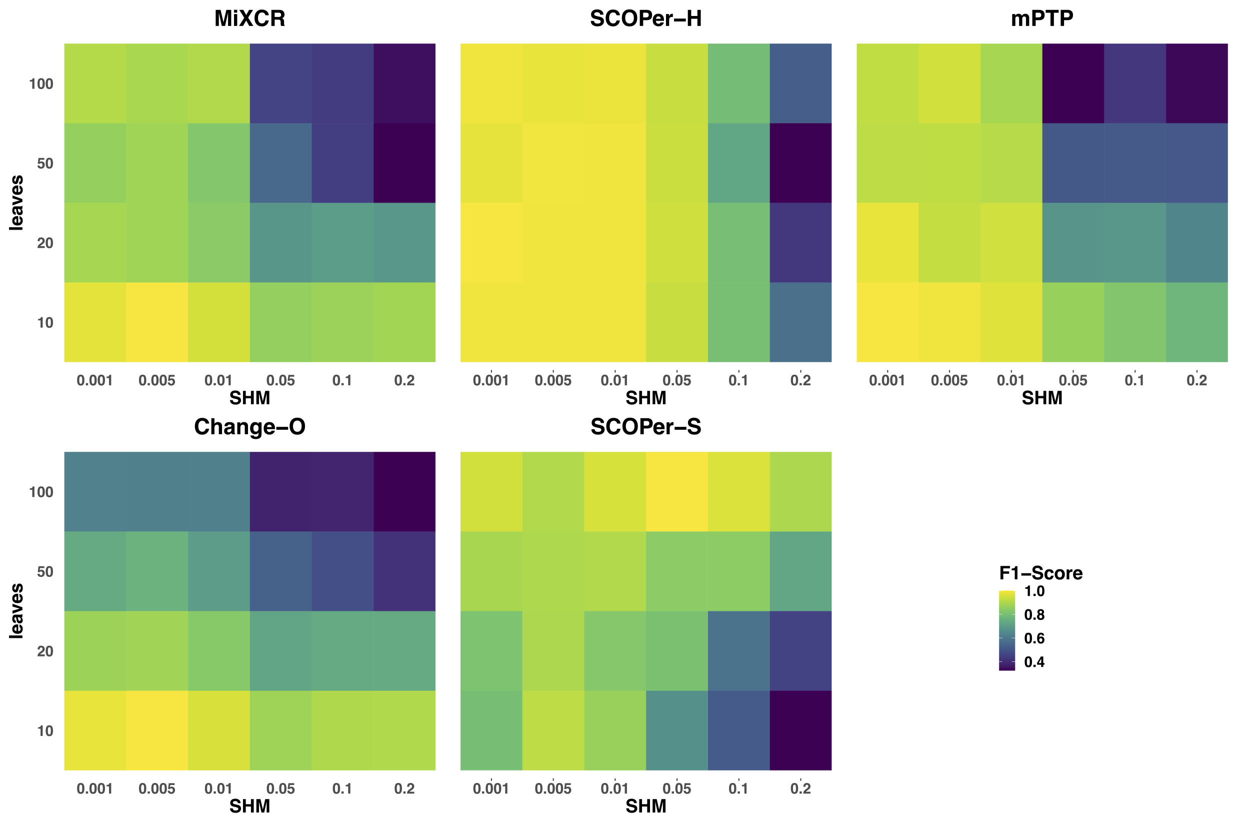

Figure S3: **F1-Score across different parameters for all methods.** For this analysis we removed singletons. The F1-score was calculated by taking the average of all simulations with the specific leaf and SHM configuration.

bioRxiv preprint doi: https://doi.org/10.1101/2024.05.29.596491; this version posted June 2, 2024. The copyright holder for this preprint (which was not certified by peer review) is the author/funder, who has granted bioRxiv a license to display the preprint in perpetuity. It is made available under aCC-BY 4.0 International license.

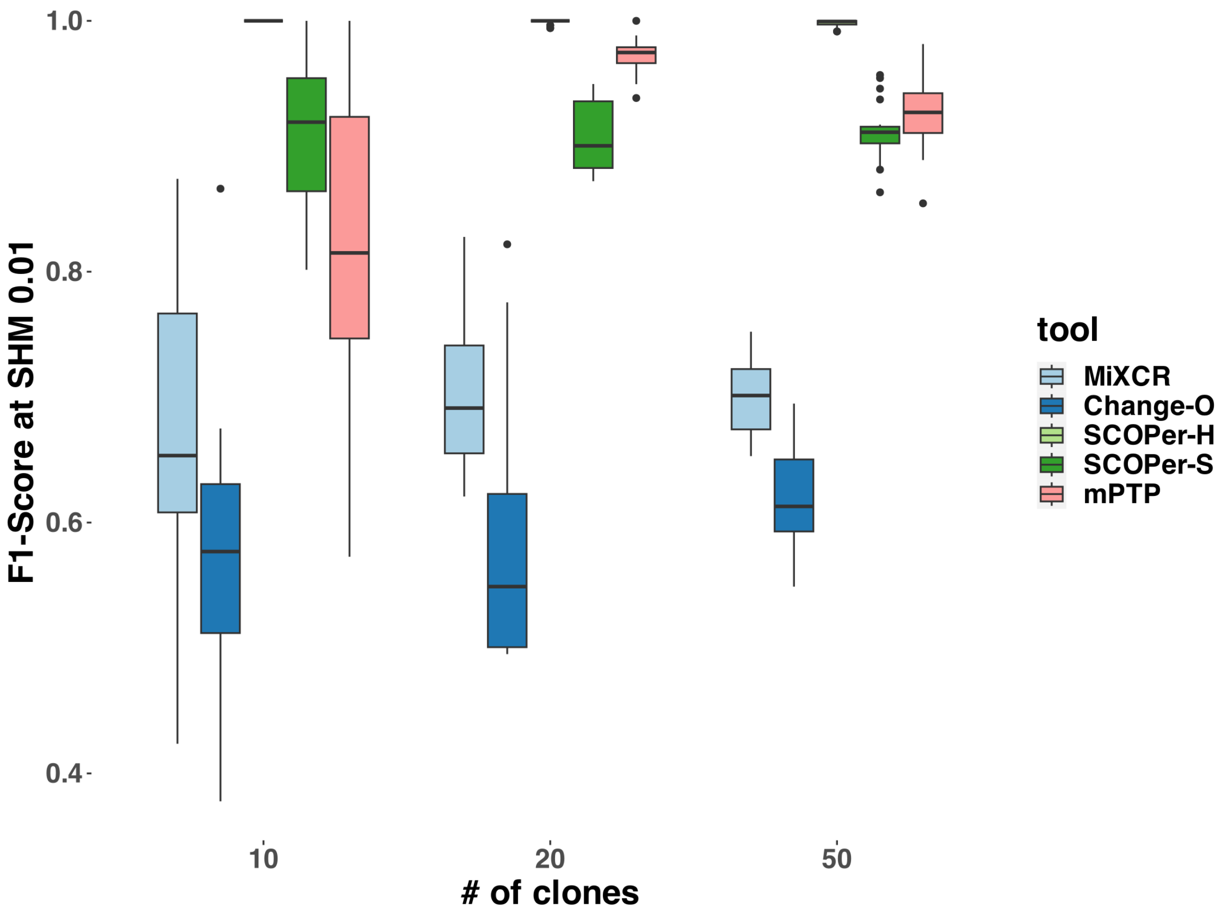

Figure S4: **F1-Score across different numbers of clones for all methods.** For this analysis we removed singletons. The F1-score was calculated by taking the average of all simulations with the same number of clones.

bioRxiv preprint doi: https://doi.org/10.1101/2024.05.29.596491; this version posted June 2, 2024. The copyright holder for this preprint (which was not certified by peer review) is the author/funder, who has granted bioRxiv a license to display the preprint in perpetuity. It is made available under aCC-BY 4.0 International license.

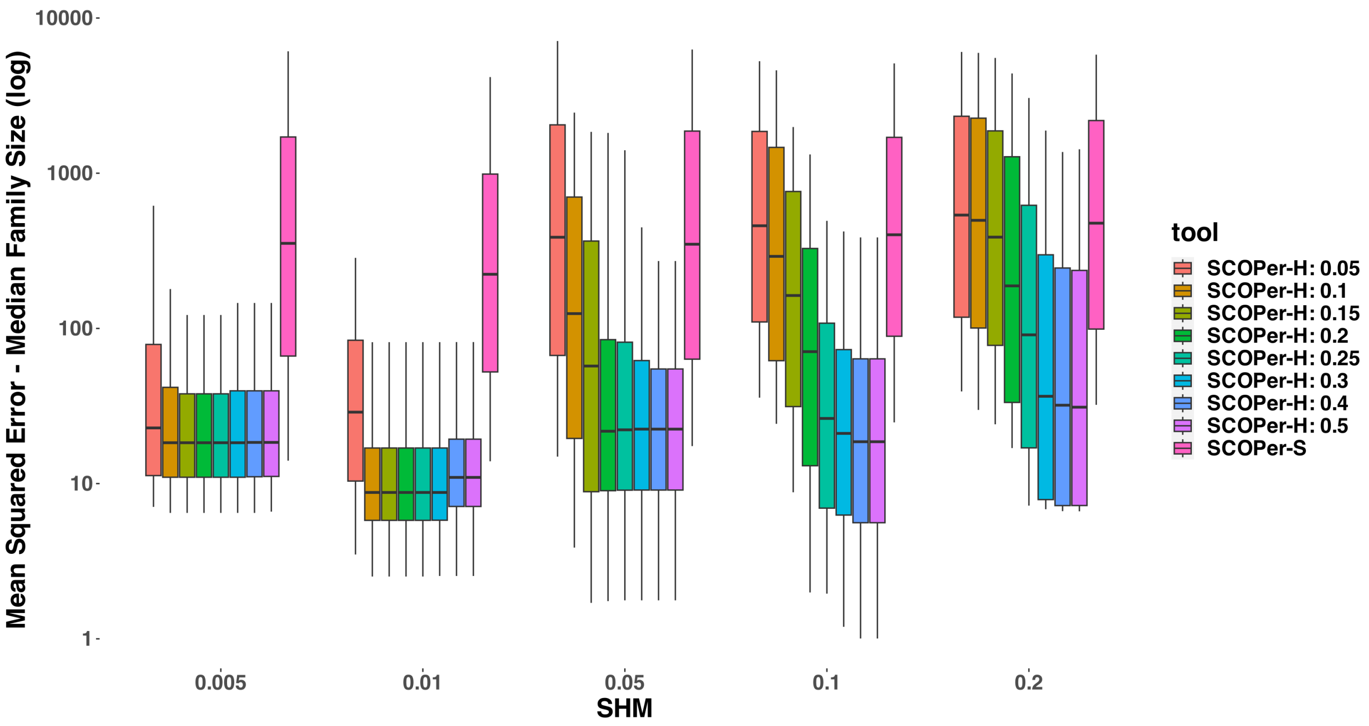

Figure S5: **MSE of the median family size for different SCOPer-H thresholds.** For this analysis we removed singletons. SCOPer-H: 0.15 is the one used in all other analyses.

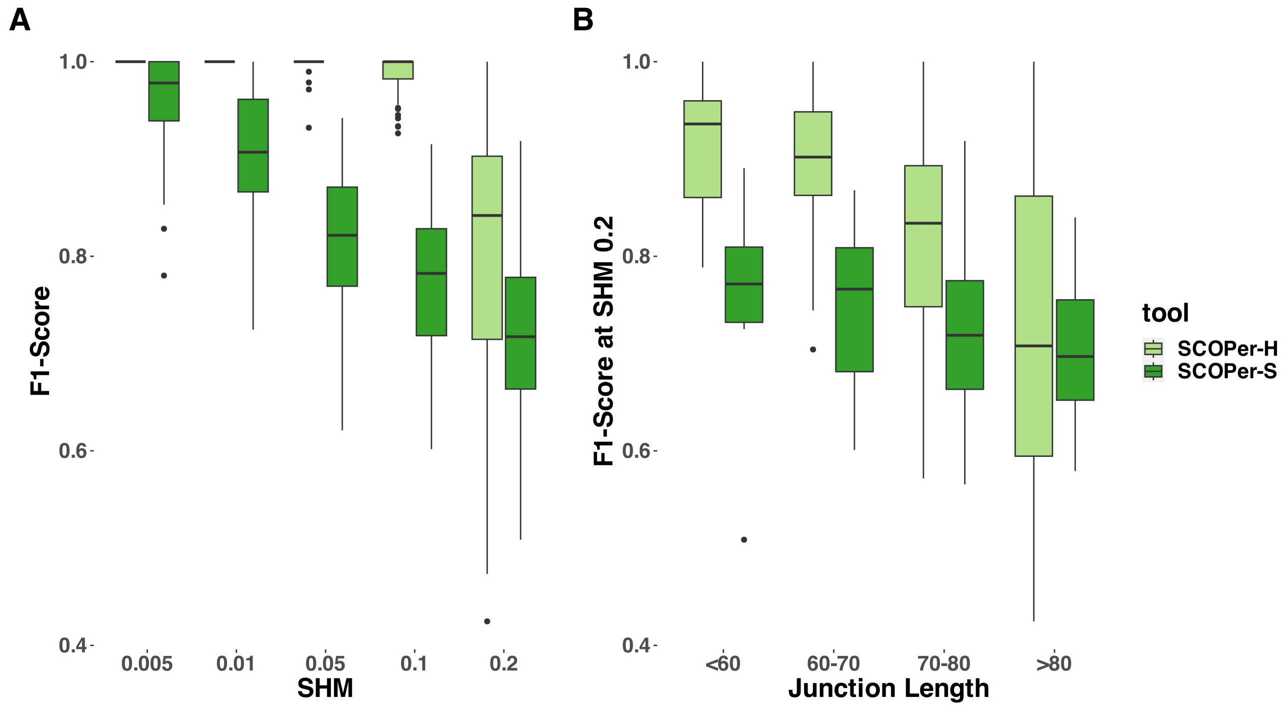

Figure S6: **F1-Score across different parameters for SCOPer-H and SCOPer-S**. For this analysis we removed singletons. A) different SHM rates B) different junction region lengths (nt). The junction region length was calculated by SCOPer.

bioRxiv preprint doi: https://doi.org/10.1101/2024.05.29.596491; this version posted June 2, 2024. The copyright holder for this preprint (which was not certified by peer review) is the author/funder, who has granted bioRxiv a license to display the preprint in perpetuity. It is made available under aCC-BY 4.0 International license.

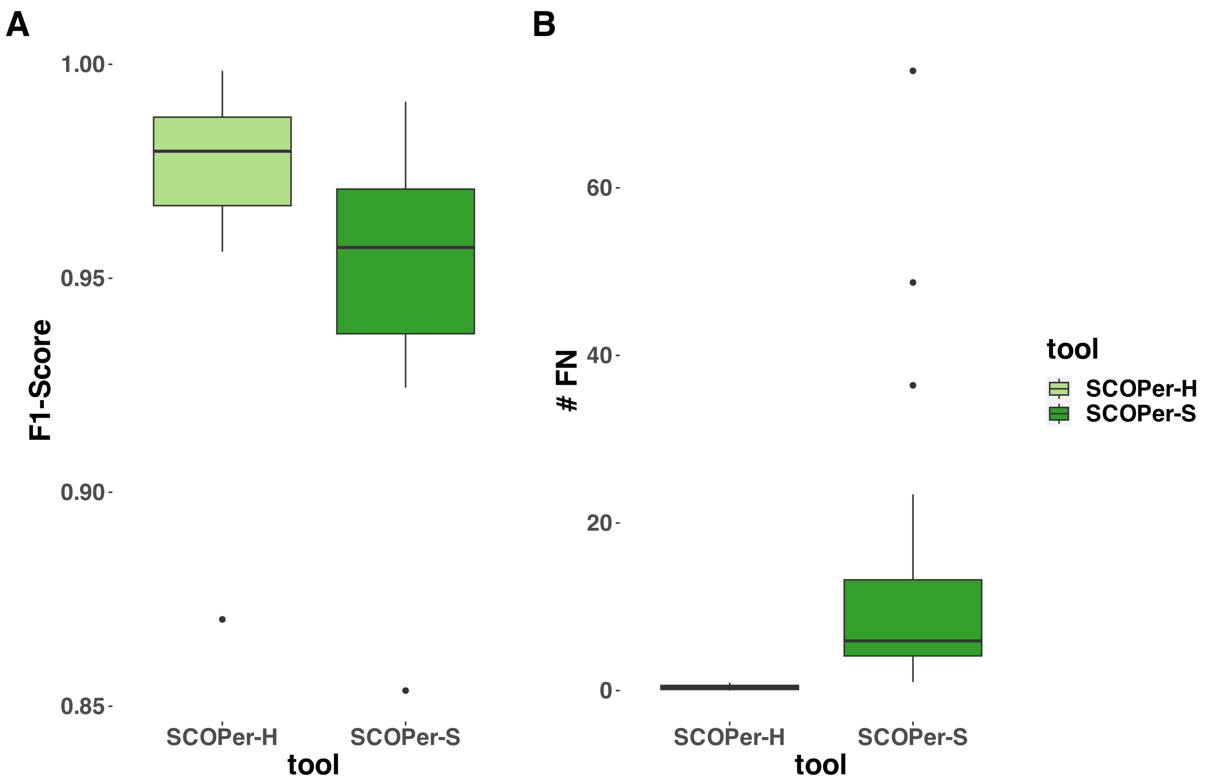

Figure S7: **Evaluation of a subset of simulations provided by Nouri et al. [11]** A) F1-score for SCOPer's hierarchical and spectral model B) number of False Negatives for SCOPer's hierarchical and spectral model. For each simulation we took the 20,000 first results and evaluated them.

bioRxiv preprint doi: https://doi.org/10.1101/2024.05.29.596491; this version posted June 2, 2024. The copyright holder for this preprint (which was not certified by peer review) is the author/funder, who has granted bioRxiv a license to display the preprint in perpetuity. It is made available under aCC-BY 4.0 International license.

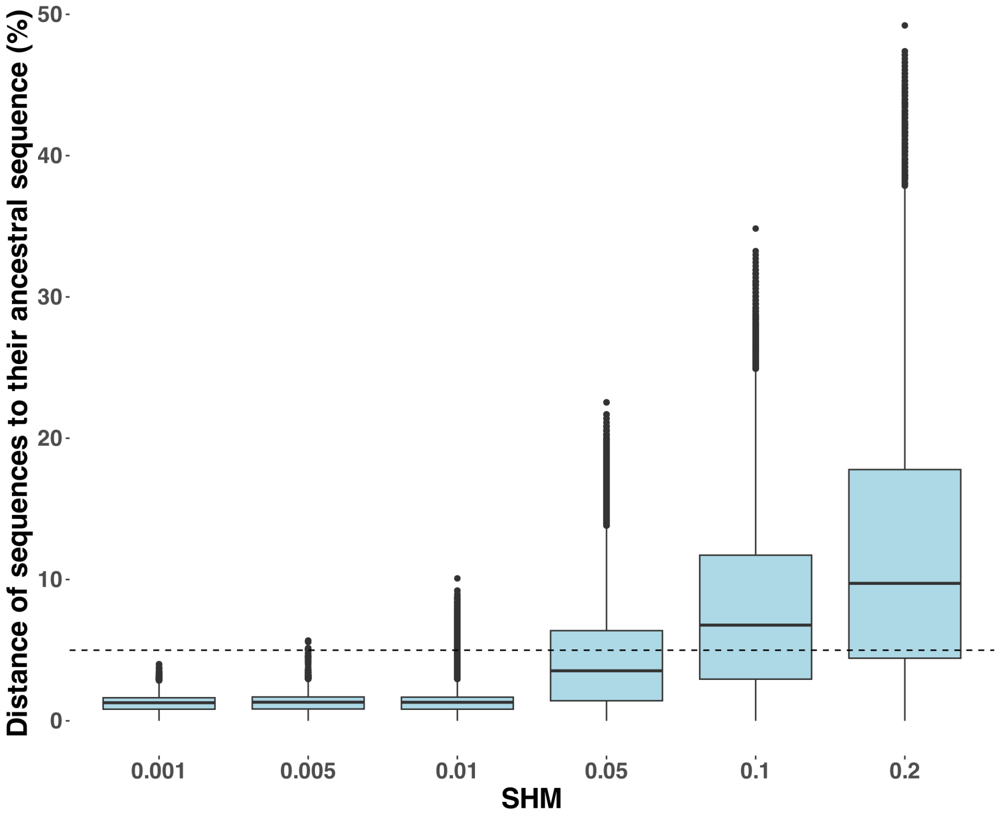

Figure S8: **Sequence Divergence between the simulated sequences and their ancestral sequence**. The dashed line is at 5%, which is a known level of divergence in real sequences.

bioRxiv preprint doi: https://doi.org/10.1101/2024.05.29.596491; this version posted June 2, 2024. The copyright holder for this preprint (which was not certified by peer review) is the author/funder, who has granted bioRxiv a license to display the preprint in perpetuity. It is made available under aCC-BY 4.0 International license.

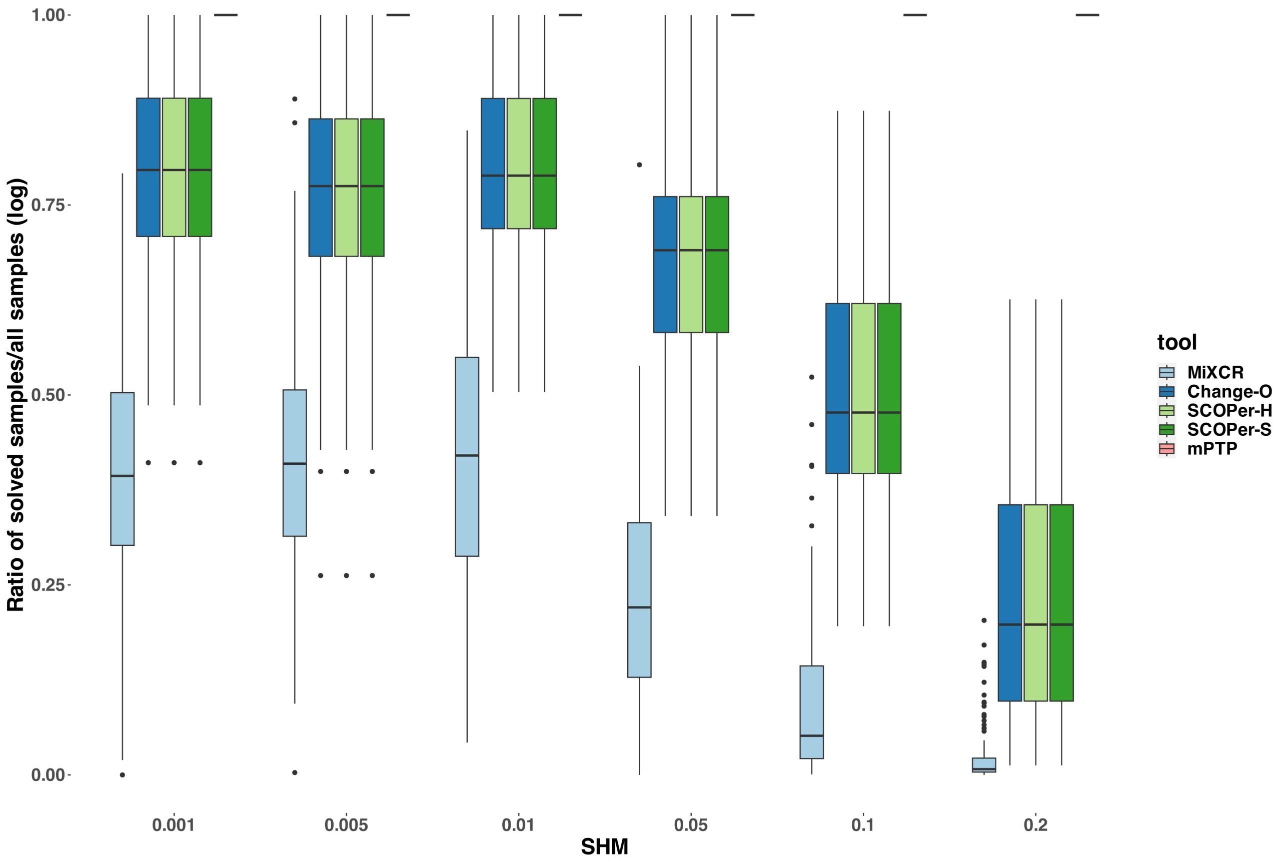

Figure S9: **Ratio of solved samples/all samples (log) across different SHM rates in simulations with "fake" V genes.** For this analysis we counted the amount of sequences that the methods assigned to a clonal family and divided that by the number of all sequences from the input.

bioRxiv preprint doi: https://doi.org/10.1101/2024.05.29.596491; this version posted June 2, 2024. The copyright holder for this preprint (which was not certified by peer review) is the author/funder, who has granted bioRxiv a license to display the preprint in perpetuity. It is made available under aCC-BY 4.0 International license.

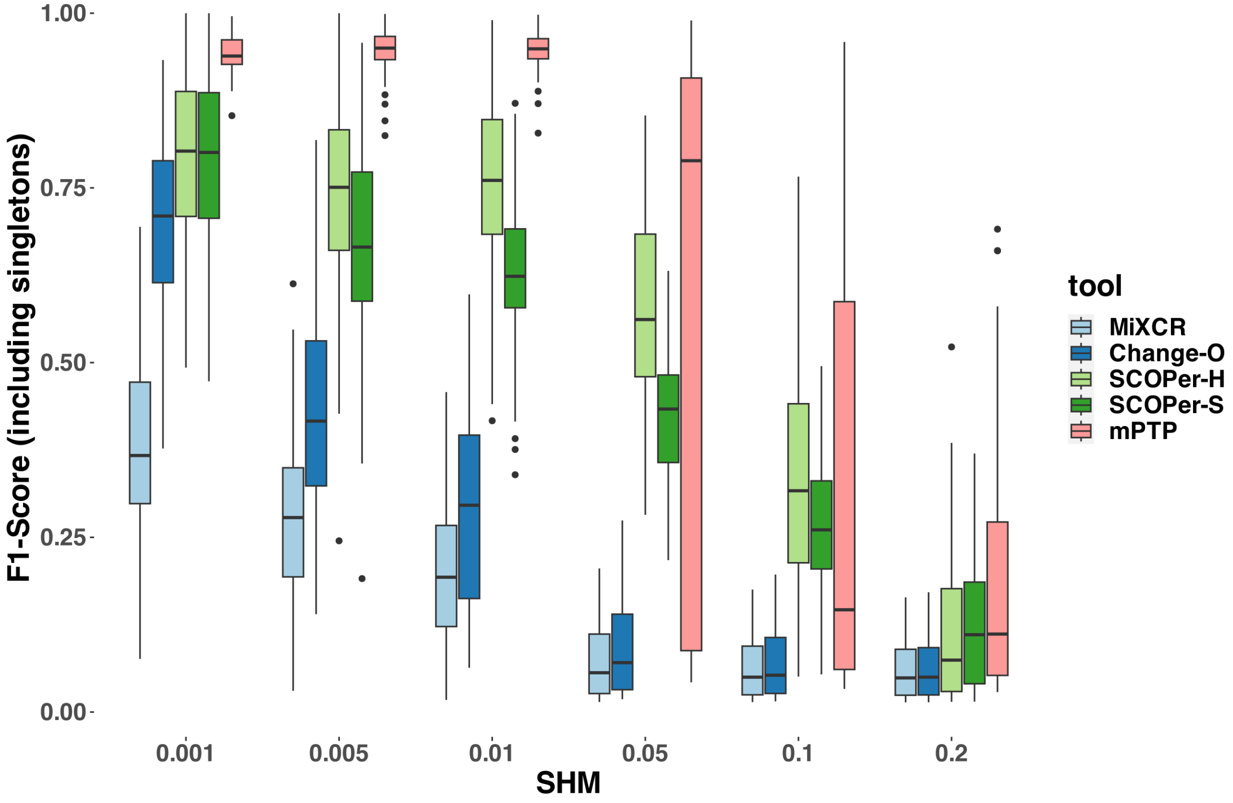

Figure S10: **F1-Score yielded by the different methods across different SHM rates for simulations with "fake" V genes (includes singletons)** The F1-score was calculated by taking the average of all simulations with the specific SHM configurations.

bioRxiv preprint doi: https://doi.org/10.1101/2024.05.29.596491; this version posted June 2, 2024. The copyright holder for this preprint (which was not certified by peer review) is the author/funder, who has granted bioRxiv a license to display the preprint in perpetuity. It is made available under aCC-BY 4.0 International license.

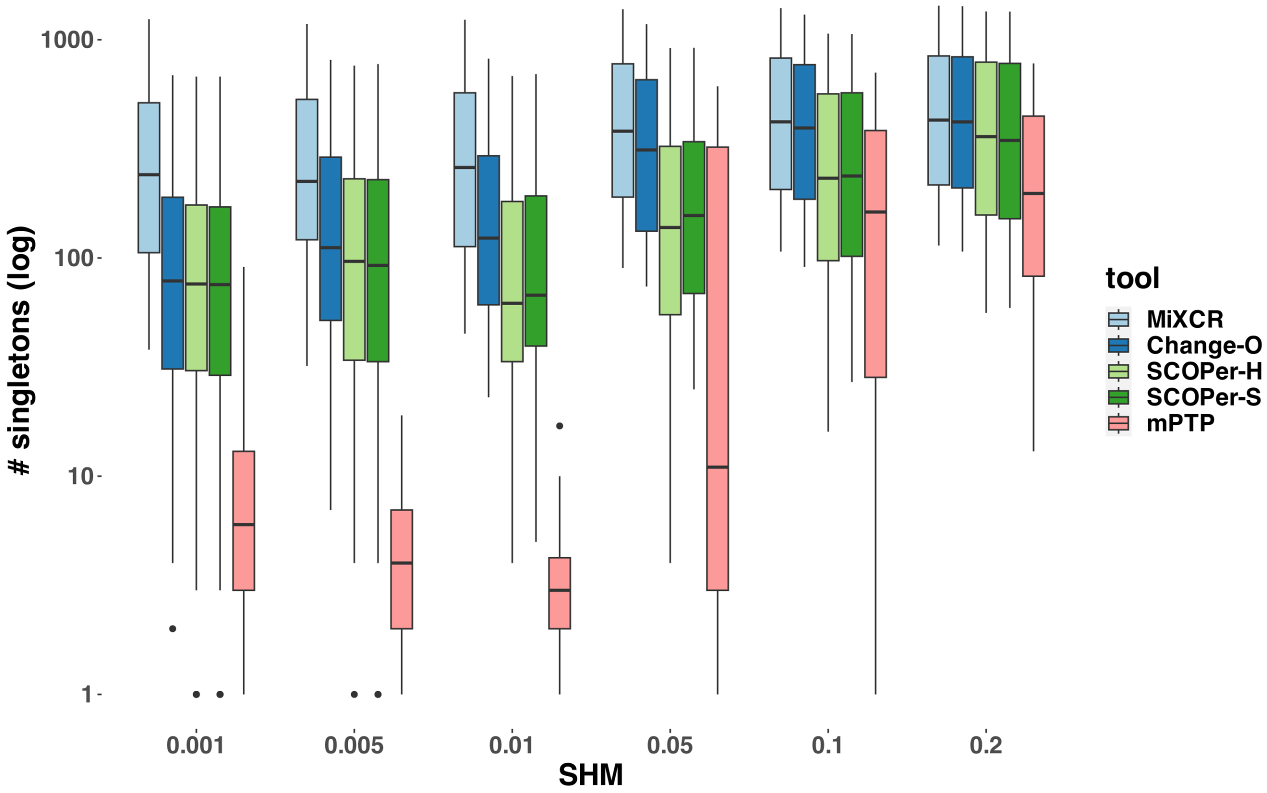

Figure S11: **Number of singletons derived by each method for simulations with "fake" V genes**

bioRxiv preprint doi: https://doi.org/10.1101/2024.05.29.596491; this version posted June 2, 2024. The copyright holder for this preprint (which was not certified by peer review) is the author/funder, who has granted bioRxiv a license to display the preprint in perpetuity. It is made available under aCC-BY 4.0 International license.

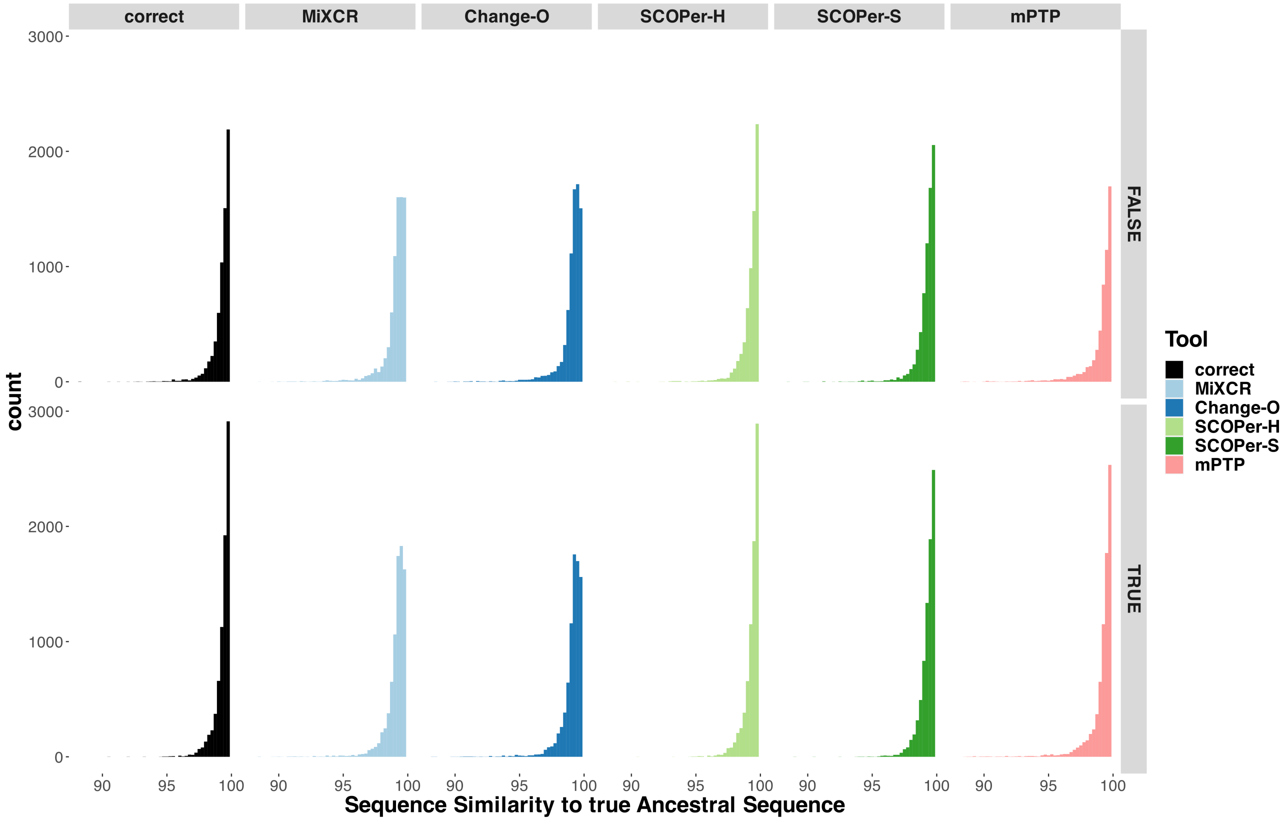

Figure S12: **Sequence Similarity between the real ancestral sequence and the derived ancestral sequence** based on the clonal families discerned by the methods split between using the unrooted tree provided by RAxML-NG (FALSE), and rooting at the midpoint (TRUE)
